# Genotypic Diversity Effects on the Performance of *Taraxacum officinale* Populations Increase with Time and Environmental Favorability

**DOI:** 10.1371/journal.pone.0030314

**Published:** 2012-02-10

**Authors:** Emily B. M. Drummond, Mark Vellend

**Affiliations:** 1 Department of Botany, Biodiversity Research Centre, University of British Columbia, Vancouver, British Columbia, Canada; 2 Département de Biologie, Université de Sherbrooke, Sherbrooke, Québec, Canada; University of San Diego, United States of America

## Abstract

Within-population genetic diversity influences many ecological processes, but few studies have examined how environmental conditions may impact these short-term diversity effects. Over four growing seasons, we followed experimental populations of a clonal, ubiquitous weed, *Taraxacum officinale*, with different numbers of genotypes in relatively favorable fallow field and unfavorable mowed lawn environmental treatments. Population performance (measured as total leaf area, seed production or biomass) clearly and consistently increased with diversity, and this effect became stronger over the course of the experiment. Diversity effects were stronger, and with different underlying mechanisms, in the fallow field versus the mowed lawn. Large genotypes dominated in the fallow field driving overyielding (via positive selection effects), whereas in the mowed lawn, where performance was limited by regular disturbance, there was evidence for complementarity among genotypes (with one compact genotype in particular performing better in mixture than monoculture). Hence, we predict stronger genotypic diversity effects in environments where intense intraspecific competition enhances genotypic differences. Our four-year field experiment plus seedling establishment trials indicate that genotypic diversity effects have far-reaching and context-dependent consequences across generations.

## Introduction

The connection between biodiversity and ecosystem functioning has become a central issue in ecology [1]–[4]. Although the role of species diversity remains controversial [5], ecosystem properties such as primary productivity (e.g. [6]), resistance to exotic plant invasion (e.g. [7], [8]) and nutrient retention (e.g. [9]) have been shown to increase with plant diversity in experimental ecosystems. Recent research has demonstrated that genetic diversity within species may also have important ecological consequences of surprisingly large magnitude (reviewed in [10]). Within a single-generation, standing genetic variation, especially in dominant or keystone species [11], may enhance plant population productivity [12]–[15] and resistance to disturbance [16], [17], promote species diversity within competitive plant communities [18]–[20], reduce susceptibility to plant invasions [21] and influence associated arthropod community composition and diversity [15], [22], [23]. Genetic diversity and identity effects on population performance may be of particular importance for exotic species, for which initial genetic diversity varies widely [24]–[26]. It is largely these short-term, ecological consequences of genetic diversity that we concern ourselves with here, independent of any longer-term effects on adaptive evolution.

That genetic diversity can affect ecological processes is now well established (see [10]), but it is less clear how diversity effects are generated and how environmental conditions may moderate their strength. Consequently, we have little predictive understanding of when and why genetic diversity effects will occur, and how important these effects are relative to other ecological factors affecting populations and communities. This gap in understanding has limited our ability to resolve discrepancies among studies. For example, while some studies have found strong effects of genetic diversity on population productivity and fitness (e.g. [14], [15], [17], [21], [27]–[29]), others have not (e.g. [30]–[32]), and the reasons for this variability remain unclear. Intuitively, the strength of genetic diversity effects should depend on the magnitude of underlying genetic variation (among individuals in relevant traits), and hence we might expect environmental conditions to modulate these effects via their influence on the expression of genetic variance (as described in [33]). Some evolutionary theorists have posited that unfavorable conditions should magnify genetic differences (as individual genotypes are pushed to their limits), while others have argued the reverse, that favorable conditions (where genotypes can develop to their full potential) might exaggerate differences (see [34] for a review of the evidence). While no theoretical consensus exists, relevant empirical studies of how environmental variables (such as soil fertility, disturbance regime, etc.) may influence genetic diversity effects are few. Purely circumstantial evidence (from a marine system) suggests that diversity effects might only be revealed under poor conditions [16], [35], [36], however this prediction has generally not borne out in experimental studies of terrestrial plants. While diversity effects were stronger in deer herbivory vs. deer exclosure treatments in a field experiment [17], there were no differences among environmental treatments in several artificial, pot-based experiments [14], [30], [37].

Here we conduct the first direct experimental field test of how genotypic diversity effects depend on environmental favorability, using asexual, clonal dandelions, *Taraxacum officinale* G. H. Weber ex Wiggers. Dandelions are a ubiquitous, perennial weed distributed throughout temperate zones of the world, often found in disturbed habitats. We created replicate dandelion populations of low (1-genotype), medium (2 genotypes) and high (4–5 genotypes) genotypic richness under field conditions, in two environmental treatments that represent common dandelion habitats, relatively favorable “fallow fields” and unfavorable “mowed lawns”. Interspecific competition and/or disturbance reduced performance in the mowed lawn in comparison with the fallow field, making it the less favorable environment. We followed populations over four growing seasons, a duration that exceeds most experiments on this topic to date. We collected data on individual plant fitness components to test (1) that genotypic diversity increases population performance, (2) that this effect is impacted by environmental favorability, and (3) the underlying mechanisms of diversity effects. Non-additive effects of biodiversity (where mixture performance is not predictable based on monocultures) may be driven by selection effects (where genotypes with particular traits rise to competitive dominance) or by complementarity (arising from niche differentiation or facilitation among genotypes) [38].

## Methods

### Study Species

While both diploid and triploid individuals of *Taraxacum officinale* occur in its native Europe, only the asexual triploids have been found in the invaded North American range [39]. Populations across the continent contain five genotypes on average (range of 1–13) [40] and genotypes have been shown to vary in ecologically important traits [41]–[43]. Dandelion genotypes used in this study (identified using microsatellite DNA markers) were collected around Vancouver (British Columbia, Canada) and shown to vary substantially in morphology and fitness components in a common garden [44]. While six putative genotypes were incorporated into our experiment, after planting it was discovered that a genotyping error had been made such that two of the genotypes were in fact the same. This had essentially no impact on our ability to test our experimental hypotheses. Our five focal genotypes represent >75% of individuals in four populations in the vicinity of our study site, with each population containing 4–5 genotypes, as in our high diversity treatment (M. Scascitelli & M. Vellend, unpublished microsatellite data).

### Study Site and Experimental Design

Our study was conducted at Totem field, a 12-ha research facility on the University of British Columbia campus (Vancouver, Canada) in which non-experimental areas are maintained as non-irrigated, regularly mown lawn, dominated by grasses including *Poa pratensis*, *Festuca* spp., and *Holcus lanatus*, the moss *Rhytidiadelphus squarrosus*, and several broad-leaved weed species. Experimental plots (n = 180) were arranged in nine rows of 20 adjacent plots (0.5×0.5 m), with 1 m borders between rows. We randomly assigned half of the plots (on a plot-by-plot basis) to be “mowed lawns” and half to be “fallow fields” (created by hand-tilling the sod). Within each environmental treatment, we created diversity treatments of 1, 2, 4 or 5 genotypes (n = 35, 25, 20 or 10 respectively; replication was uneven due to the genotyping error) (Table S1), randomly assigning each plot a diversity treatment. Thus, both factors, environment and diversity, were completely randomized. We planted populations of 10 dandelion seedlings (pre-established in partially shaded pots at Totem) in each plot in June 2007, with all seedlings of a single genotype in 1-genotype plots, five seedlings of each genotype in 2-genotype plots, and so on. To permit individual identification, seedlings were planted in a grid formation (rows with 2, 3, 3, and 2 seedlings), with 10 cm between individuals, resulting in a density of 185 plants per m^2^ which represents the upper-end of observed densities in the Vancouver area. Seedlings that died in the first 6 weeks after planting (<5%) were replaced.

### Experiment Maintenance and Response Variables

During early establishment (June to August 2007), fallow field plots were weeded to minimize plot-to-plot variation (in growing conditions for dandelions) and mowed lawn plots were hand clipped weekly (to sod-level), avoiding the dandelion seedlings to enhance survival. For the rest of the experiment (August 2007–May 2010), clipping was done monthly during the growing season in the mowed lawn (and included the dandelions) and non-planted dandelion individuals were weeded regularly. For the first two growing seasons (in 2007 & 2008), we recorded individual plant performance using two correlated measurements: total leaf area (measured monthly, just prior to clipping in the mowed lawn plots), and seed number (cumulative). Leaf area was estimated as LA = 0.221×N×L×2W (Multiple Linear Regression: R^2^ = 0.95, n = 56 field-collected plants) [44], where N is the number of leaves >4 cm long, L is the length of the longest leaf, and W is the maximum distance from the mid-vein to a leaf lobe tip on the longest leaf. Total seed number was calculated as the observed number of seed-heads multiplied by the mean number of seeds per seed-head. Genotype (G), environment (E) and season (S) specific averages (n = 392 field-collected seed-heads) were used as these three factors interacted in a generalized linear model predicting seed number as a Poisson variable (G×S: p = 0.01, S×E: p = 0.02, G×S×E: p = 0.06). For the last two growing seasons (2009 & 2010), individuals could no longer be reliably distinguished and so we recorded the cumulative number of seed-heads *per plot*. The experiment was harvested in May 2010 and the per plot aboveground biomass was determined by oven drying (until constant weight) all dandelion material.

### Seedling establishment experiment

In order to predict the cross-generation effects of observed variability in seed production, we conducted a field trial to estimate differences in establishment success from seed for each genotype, in each environment. We used a split-plot design, with environment (n = 5 for each) randomly assigned to 10 whole-plots (24×48 cm) and genotype (n = 1 for each of the five genotypes plus a no-seed control) randomly assigned to six sub-plots (8×18 cm) per whole-plot. Whole-plots were created in a single row alongside the main experiment at Totem field in May 2008, and 50 seeds of a given genotype were sown into each sub-plot. Germinants were counted regularly over the next five months, on a timeline commensurate with emergence rates.

### Data Analysis

To test for effects of environment, diversity (i.e. genotypic richness) and their interaction, we performed separate analyses of covariance (ANCOVAs) on the leaf area (six samples between 2007 & 2008), seed number (cumulative 2007–2008), seed head (cumulative 2009–2010) and biomass (2010) data, summed for all plants in a plot. (The complex variance-covariance structure in the leaf area data precluded a repeated-measures analysis.) Diversity was treated as a categorical variable with three levels (low, medium and high), and plant performance data were transformed as needed to meet model assumptions (Table S2). The first principal component, prin1, from a principal components analysis on vegetation composition data collected prior to the experiment (Table S3), was included as a covariate to account for spatial variability in the edaphic environment. Prin1 explained 28% of the variation in vegetation composition, and was negatively correlated with dandelion performance in both environments.

For the individual-level performance data (2007–2008 leaf area & seed number), we used the additive partition of Loreau & Hector [38] to test for underlying mechanisms. We calculated net biodiversity (ΔY), complementarity, and selection effects separately for each environment, after first correcting the raw data to account for variability in prin1, by regressing plot productivity on prin1, and then adding the residuals to the mean productivity. To test if the mean effects differed from zero, we first pooled the mixtures across richness levels, as there was no relationship between richness and effect size (Linear Regression: p>0.05). T-tests were used when the data were normally distributed, or could be transformed (Table S4), and the more conservative, distribution-free sign-test was used in severe cases of non-normality.

As the strength of genetic diversity effects is expected to depend on the magnitude of variation among genotypes in key traits, we also used the individual-level performance data to calculate the coefficient of variation (CV) among genotypic means separately for each environment, and in monoculture versus mixture. We also compared the variance among genotypic means in mixture versus monoculture (by environment), and used F-tests to assess whether or not the ratio of the variances was greater than one. Ratios greater than one suggest that inter-genotypic interactions in mixtures exaggerate size differences.

For all genotypes and in both environments, the number of seedlings observed increased sharply to a plateau (after about a month) and then gradually declined, likely as a result of self-thinning. Hence, we examined the effects of genotype, environment and their interaction on the maximum number of seedlings, using restricted maximum likelihood (REML) in a mixed model. While environment and genotype were treated as fixed effects (as we selected these objectively), the effect of whole-plots was random. The significance of the fixed effects was determined using an F-statistic with the degrees of freedom approximated using the Satterthwaite method.

Statistical analyses were performed in SAS, version 9.1 (SAS Institute, North Carolina, USA), with simple statistics obtained in R, version 2.7.0 (R Development Core Team 2008).

## Results

### Both environment and diversity shape population performance

**Figure 1:**
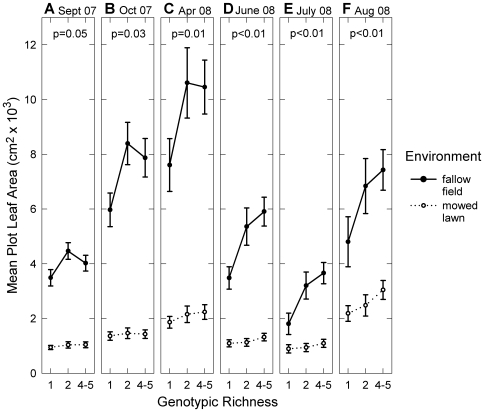
Effects of richness and environment on plot performance over time. Mean plot leaf area (cm^2^) ±1 SE versus genotypic richness, in two environmental treatments (N = 180), for six measurement dates from Sept. 2007 to Dec. 2008 (a–f). Richness was treated as a categorical variable with three levels: low (1 genotype), medium (2 genotypes), and high (4–5 genotypes). There was a significant effect of environment at all dates (ANCOVA on transformed data: p<0.0001) and of richness at most dates (see p-values in Figure). In July 2008 (e), there is also a significant richness-by-environment interaction (p = 0.04).

**Figure 2:**
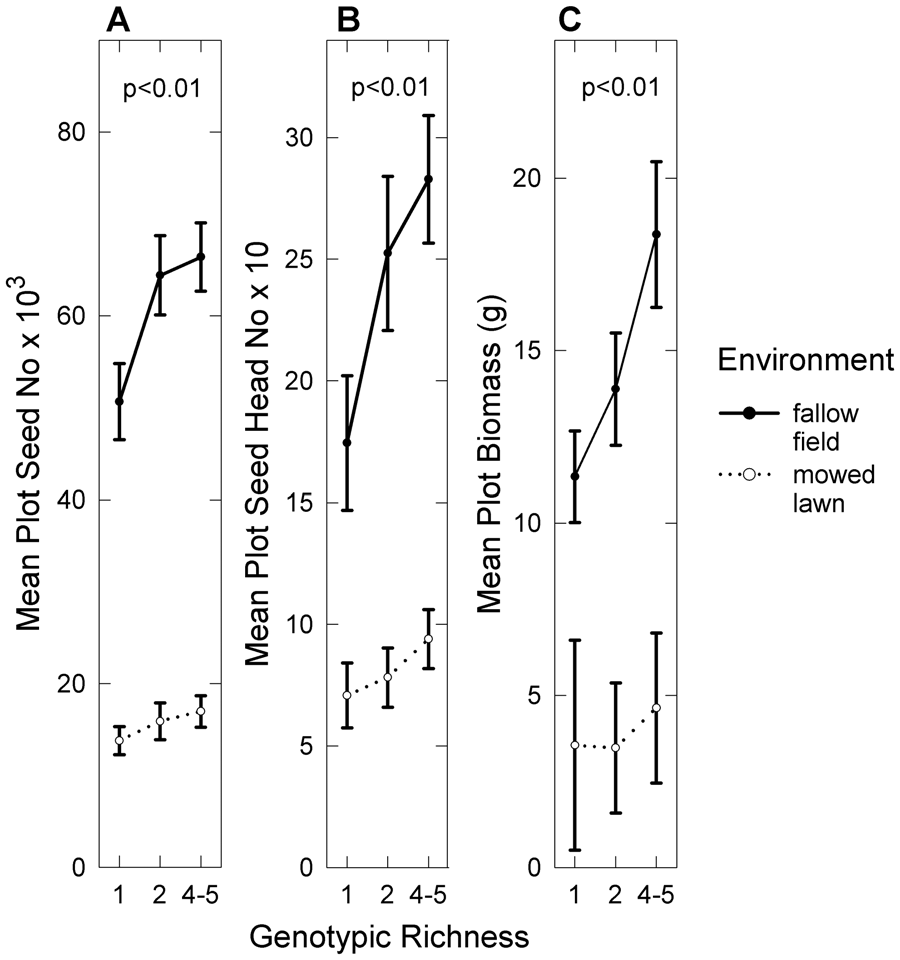
Effects of richness and environment on cumulative plot performance. Mean plot (a) cumulative seed number (2007–2008), (b) cumulative seed head number (2009–2010) or (c) aboveground biomass (g) (2010) ±1 SE versus genotypic richness, in two environmental treatments (N = 180). Richness was treated as a categorical variable with three levels: low (1 genotype), medium (2 genotypes), and high (4–5 genotypes). For all variables, there was a significant effect of environment (ANCOVA on transformed data: p<0.0001) and of richness (see p-values in Figure).

Increasing diversity strongly and consistently enhanced population performance throughout the experiment (Figure 1, Figure 2, Table S2), whether performance was measured as leaf area (36% significant increase from low- to high-diversity plots), seed (27%) or seed head (32%) production, or final biomass (31%). Population performance was also significantly higher in the fallow field compared to the mowed lawn (leaf area: 283% increase; seed no: 286%; seed head no: 188%; biomass: 270%), as we predicted given that the fallow field was the more favorable environment (due to lower levels of interspecific competition and no disturbance). The environment-by-diversity interaction was not significant (p>0.05), except for leaf area in July 2008. We did not correct our results for multiple comparisons, and so interpret only general trends and not single p-values.

### Diversity effects grow stronger over time

The strength of the diversity effect increased over the course of the first two years, as shown by increasing F-values (see leaf area results in Table S2) and greater gains (with diversity) in plot leaf area over time. The increase in mean leaf area from low- to high-diversity plots went from 13% to 48% (first to last time point). Meanwhile, the effects of environment and the covariate (prin1) both decreased over time (see F-values in Table S2). The shape of the relationship between population performance and diversity also shifted from a non-linear to a linear relationship (see Figure 1 & Figure 2). Post-hoc tests revealed that medium-diversity means, while significantly different from low-diversity means in the 2007–2008 data (Tukey's HSD: p<0.05), were no longer different in the 2009–2010 data.

### Different mechanisms drive diversity effects in different environments

**Figure 3:**
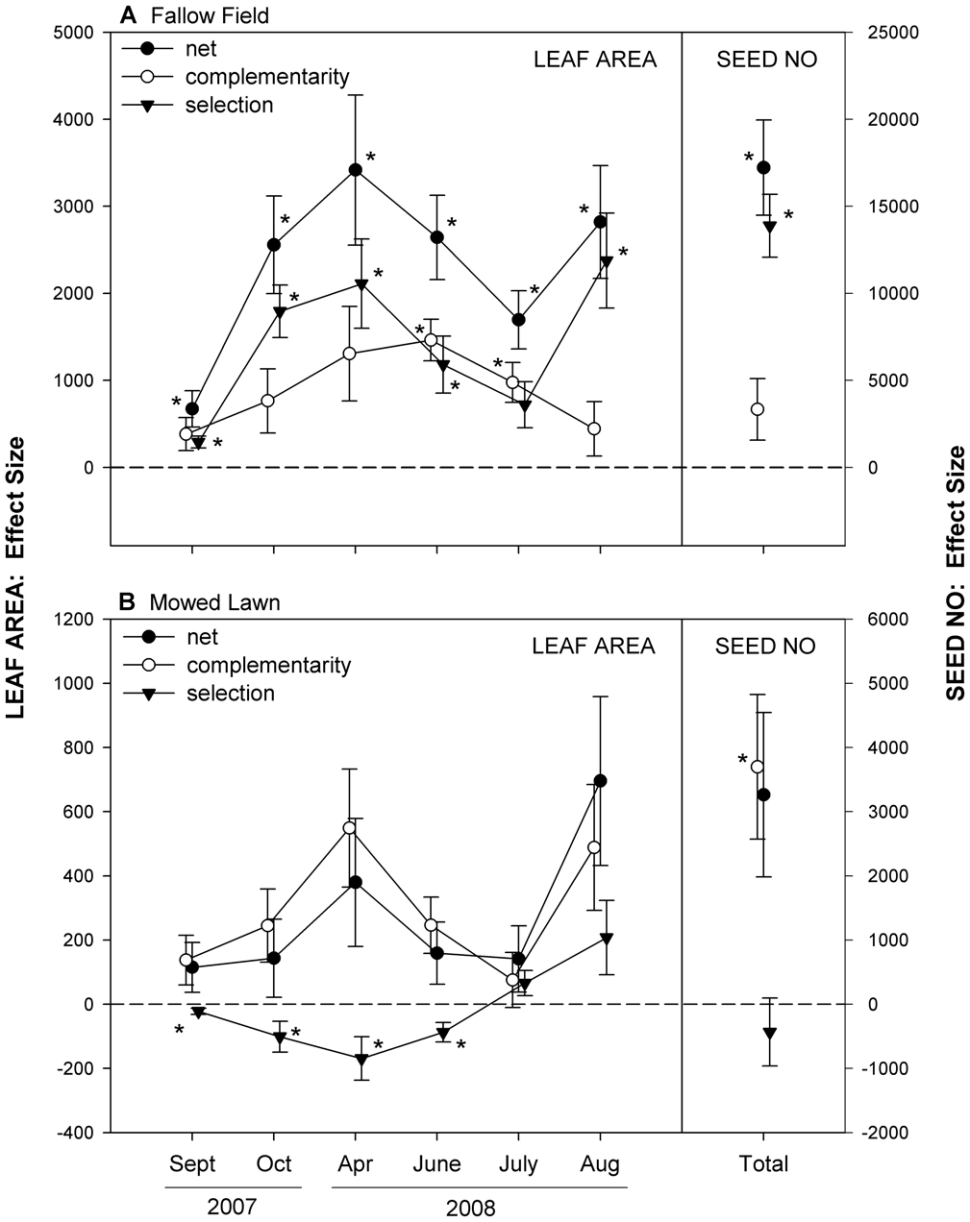
Partitioning of net biodiversity effects. Mean net biodiversity (•), complementarity (○) and selection effects (▾) ±1 SE for plant leaf area (cm^2^) over time and cumulative seed number (2007–2008) (N = 55). Means are shown separately for (a) the fallow field and (b) the mowed lawn. The dashed line indicates an effect size of zero. A star (*) indicates that the mean is significantly different from zero (p<0.05, t-tests where data were normal or could be transformed, sign-tests for remaining cases: see text for details).

**Figure 4:**
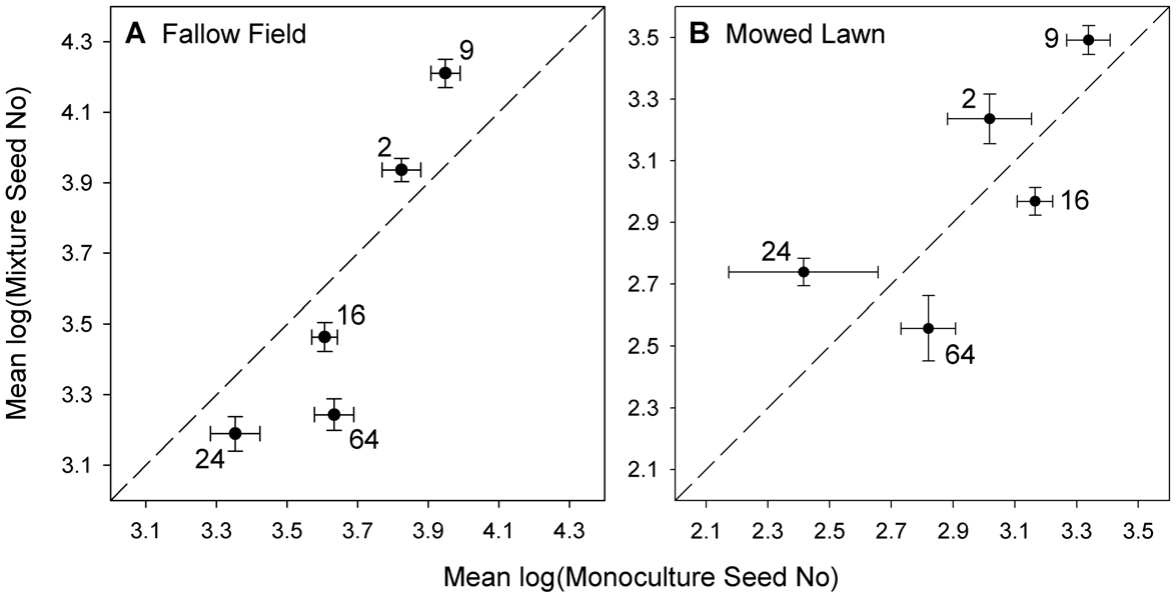
Comparison of genotypic performance in mixture versus monoculture. Dandelion genotype mixture (plot genotypic richness >1) versus monoculture (plot genotypic richness = 1) means ±1 SE for cumulative seed number (2007–2008) (N = 1800). Means are shown separately for (a) the fallow field and (b) the mowed lawn. Seed number was log-transformed before the means were calculated for each genotype. The dashed line indicates a 1∶1 relationship. Numbers refer to specific genotypes.

Net biodiversity effects and their complementarity and selection components (calculated sensu [33] for leaf area and seed number in 2007–2008) revealed differences in how diversity affected performance in the two environments (Figure 3). In the fallow field, net biodiversity effects were universally positive and significantly greater than zero (Table S4), indicating that average genotypic performance was higher in mixture versus monoculture. This effect was largely driven by a positive selection effect; genotypes 2 and 9, the two best genotypes in monoculture, performed better in mixture than monoculture (Figure 4, Figure S1). In contrast, the net biodiversity effects in the mowed lawn were much smaller and, while positive, were not significantly so. Here, genotypes 24, 2 and 9 (low, medium and high performance in monoculture) performed best in mixture resulting in a positive complementarity effect; this effect tended to be cancelled out by a negative selection effect (due to genotype 24 getting the most benefit from growth in mixture).

### Variability in genotypic performance is enhanced in the fallow field

Differences among dandelion genotypes for leaf area (monoculture and mixture) and seed production (mixture only) were greater in the fallow field than in the mowed lawn (Table S5). For a given environment and response variable, the CV among genotypes was also generally greater in mixture versus monoculture, a difference that was more exaggerated in the fallow field relative to the mowed lawn (i.e. the percent difference in [mixture vs. monoculture] CVs was 108% vs. 33% for seed number and 42±9% vs. 35±16% for leaf area). Similarly, variance ratios (mixture/monoculture) were higher in the fallow field versus the mowed lawn for five out of seven comparisons; the ratios were at least marginally greater than one in the fallow field (Seed no: p = 0.04; Leaf area: p = 0.09), but were not different from one in the mowed lawn (Seed no: p = 0.13; Leaf area: p = 0.15).

### Seedling establishment success varies with environment and genotype

Genotypes varied in the maximum number of established seedlings in the fallow field, but not in the mowed lawn (Figure S2). This genotype-by-environment interaction was significant (Mixed Model: F_4,32_ = 3.47, p = 0.02), as were main effects of genotype (F_4,32_ = 3.72, p = 0.01) and environment (F_1,8_ = 15.9, p = 0.004). In the fallow field, genotypes 2 and 16 produced more seedlings on average than genotype 24, while all genotypes did equally poorly in the mowed lawn. The absolute number of established seedlings for any genotype was always highest in the fallow field.

## Discussion

While a wealth of recent research has clearly demonstrated important ecological consequences of genetic diversity, few studies have examined how genetic diversity effects compare to and depend on other ecological factors (but see [14], [17], [30], [36], [37]). Our study revealed strong, consistent effects of genotypic diversity and environmental favorability on dandelion population performance in a four-year field experiment (Figure 1, Figure 2). While diversity effects were comparable in magnitude with other population-level studies (roughly a 30% fitness increase, as found in e.g. [12], [16], [28], though there is considerable variation, e.g. see Discussion in [15]), and increased with time, these effects were dwarfed by the effect of environmental favorability (300% fitness increase between environments). More interestingly, we found a strong qualitative difference in the diversity effect between our two environments, with the greater effect in the more favorable environment, where plants were larger and intraspecific competition was presumably highest.

### Environmental differences modulate the strength of diversity effects

Two lines of evidence support a greater genotypic diversity effect in the fallow field, despite the generally non-significant interaction (diversity×environment) in the plot-level analysis. First, the gain in plot performance with diversity, from low- to high-diversity plots, was universally higher in the fallow field (vs. the mowed lawn). These gains were 52% vs. 20% for leaf area, 31% vs. 23% for seed number, 62% vs. 32% for seed head number, and 62% vs. 30% for aboveground biomass. Second, net biodiversity effects were significantly positive in the fallow field (i.e. diversity effects were non-additive and unpredictable based on monocultures), but not in the mowed lawn (Figure 3). This novel experimental result contrasts with circumstantial and experimental evidence showing stronger diversity effects on performance owing to stress or disturbance (e.g. deer grazing [17], goose grazing event [16], heat wave [35], winter stress [36]), and with some pot experiments that found no difference in diversity effects among environmental treatments (soil fertility [30]; density manipulations [37]; density, fertility, & herbivory manipulations [14]).

The context-dependency of biodiversity-ecosystem functioning effects has long been recognized in the species level literature [45]. The relationship between species diversity and productivity (both the shape and direction) can vary with the experimental system [1], [45], season in nature ([46] and references therein), presence or absence of other trophic levels (e.g. [47], [48]), and with varying levels of spatial or temporal heterogeneity (e.g. [49], [50]), as well as with other environmental variables. However, despite this variability and the many other ecological factors influencing productivity, diversity effects are still strong enough to produce patterns in real ecosystems [49], [51]. Interestingly, though there are still too few studies to generalize, several studies have found that species diversity effects grew stronger as resource availability increased (e.g. [52]–[56]), which likely created more opportunities for facilitation or complementarity among species. On the other hand, some studies have suggested that positive interactions among species may be greatest under stressful or disturbed conditions (e.g. [57]–[61]). In our study, the fallow field environment may have had higher resource availability, at least initially (owing to the initial absence of competing species), while the mowed lawn experienced regular disturbance.

Genotypic diversity effects are expected to be stronger when variation among genotypes (in ecologically relevant traits) is greater [10]. There is some evidence that the larger size of and the stronger competition among genotypes in our fallow field enhanced genotypic differences (Table S5). Dandelions in the fallow field grew rapaciously and quickly became intertwined aboveground, suggesting relatively strong intraspecific competition. In contrast, intraspecific competition was likely weaker in the mowed lawn, as dandelions grew in a matrix of other species and were kept small by regular clipping. This suggests that genotypic diversity effects on performance may be greatest in environments where plants reach a large size (filling the available space) and intraspecific competition is particularly acute (e.g. with high density). This effect was likely not seen in the pot experiment of Crawford & Whitney [37] due to compensatory growth in the low-density treatments (meaning that plants in low and high density pots experienced similar levels of intraspecific competition), or in our previous dandelion experiment [31] conducted under artificial conditions where nutrients were not limiting, and hence belowground competition relatively unimportant. It is worthwhile noting that the greatest effects of genotypic diversity found so far (reviewed in [10]) occurred in dense stands of habitat-forming species such as *Zostera marina* [16] and *Populus tremuloides* [62], though these dominant species may have relatively large amounts of intraspecific trait variation to begin with (see Discussion in [15]).

### Selection and complementarity effects

Testing for the underlying mechanisms in genotypic diversity studies can be difficult, as it requires identifying individual clones, which may be morphologically indistinguishable. Consequently many, but not all, studies of ecological diversity effects have had limited ability to infer underlying mechanisms (e.g. [14], [16], [21], [37]). Our experiment allowed us to explicitly test for mechanisms, at least for the first two years. Non-additive biodiversity effects in the fallow field were largely driven by positive selection effects (Figure 3, Figure 4), as the two most productive dandelion genotypes were able to dominate and suppress other genotypes in this environment. This result was not unexpected given the tenfold variation in fitness among our genotypes when grown in a common garden [44] and also given the many similar examples in the literature, particularly in agricultural studies (e.g. [13]). In contrast to the fallow field, positive interactions among genotypes were evident in the mowed lawn, where regular disturbance equalized performance (see Table S5) and led to overyielding in genotypes of different sizes (e.g. genotypes 24, 2 and 9 in Figure 4). Complementarity among genotypes has been found in several experimental systems (e.g. [15], [17], [27], [28]), including in seagrass beds recovering from a heat wave [35] where a complementarity effect outweighed a negative selection effect (as in our mowed lawn). There, poor performing seagrass genotypes in monoculture experienced reduced mortality in mixture, and the best monoculture genotype had only average performance in mixture. While we did not test for the specific processes underlying complementarity in our study, differences in flowering time (indicating differences in the timing of resource demands, as seen in [43], [44]) or differences in pathogen susceptibility are plausible.

### The temporal dynamics of biodiversity effects

Few experiments have examined genotypic diversity effects over a multi-year timeframe and none, prior to our study, have done so in terrestrial plants under field conditions. Our study found increasingly strong diversity effects over time (Table S2), while the effects of the environmental treatment and covariate decreased. Increasing competition in the fallow field treatment, both among dandelions given their rapid growth (see above), and with colonizing species, likely contributed to the reduced difference between environmental treatments over time. Our results extend to the genetic level findings of increasingly strong effects of plant species diversity on productivity over time [4], [63] (owing to species complementarity). To date, only four genotypic diversity studies have measured a change in diversity effects over time, two finding a decrease [16], [30] and two an increase [35], [36].

Differences in individual fitness among genotypes in our diversity treatments need not translate into a change in genotypic composition in the next-generation, if, for example, seed number is negatively correlated with seed viability or seedling emergence rates. However, equal seedling emergence among genotypes in the mowed lawn (Figure S2) suggests that observed differences in seed production should carry over into the next generation in this environment. In the fallow field, seedling emergence varied by genotype, with greater emergence in the three largest genotypes. By multiplying the average number of seeds per plant by the mean number of seedlings (germination trials), we obtained a rough estimate of the expected relative number of descendants. The rank order of predicted seedlings in the next generation by genotype (from most to least: 9, 2, 16, 64, 24) was nearly identical to that for seed production (9, 2, 16, 24, 64), suggesting that fitness differences should carry over, with the selection against poor-performing genotypes 24 and 64 exacerbated.

### Conclusions

Genotypic diversity clearly enhances population performance, although to varying degrees depending on environmental conditions, and our results highlight that this is more than just a transient dynamic. Fitness consequences may be enduring (over multiple generations) and of major ecological importance, given the magnitude of the effects. While previous pot-based experiments have found no effect of environmental factors on genotypic diversity effects, our field experiment reveals that, under natural conditions, diversity effects may depend on the frequency of disturbance and strength of intraspecific competition (which can be mediated by environmental variables). Even though the diversity effect (and size- and fitness-related genotypic variance) was greater in the fallow field, evidence for complementarity in the mowed lawn suggests that different kinds of phenotypic differences (i.e. in unmeasured traits) among genotypes were manifested only in this unfavorable environment. A more complete analysis of trait differences among genotypes, when grown in monoculture versus mixture, may help further elucidate how diversity effects differ in our two environments.

## Supporting Information

Figure S1 **Comparison of genotypic performance in mixture versus monoculture over time.** Dandelion genotype mixture (plot genotypic richness >1) versus monoculture (plot genotypic richness = 1) means ±1 SE for leaf area (cm^2^) at each of six measurement dates (N = 1800). Means are shown separately for a) the fallow field and b) the mowed lawn. Leaf area measurements were log-transformed before the genotypic means were calculated. The dashed line indicates a 1∶1 relationship. Numbers refer to specific genotypes.
(PDF)

Figure S2 **Maximum number of emerged seedlings by genotype and environment.** Mean of the maximum number of emerged seedlings (minus the number of emerged seedlings in a control) ±1 SE for each genotype (genotypes are designated by numbers). Means are shown separately for (A) the fallow field and (B) the mowed lawn. There was a significant genotype-by-environment interaction (Mixed Model with Satterthwaite correction, p = 0.02), and significant main effects of genotype (p = 0.01) and environment (p = 0.004). Different letters indicate significant differences within an environment (Tukey-Kramer test, p<0.05).
(PDF)

Table S1 Description of diversity treatments as completed. The numbers in the composition column refer to specific genotypes.
(PDF)

Table S2 The effects of environment, diversity (i.e. genotypic richness), and their interaction, as well as a covariate, prin1, the first axis of a principal components analysis that represented spatial variability in the edaphic environment, on multiple measures of population performance (summed across all plants in a plot): leaf area (cm^2^), seed number, seed-head number and aboveground biomass. Separate analyses of covariance (ANCOVAs) were performed for each variable and date.
(PDF)

Table S3 Species recorded in the 2007 census of plot composition. Abundance classes were used for the principal components analysis.
(PDF)

Table S4 Results of one-sample tests to determine whether mean net biodiversity, complementarity, or selection effects differed from zero. T-tests were used for net biodiversity and complementarity effects, and sign-tests for selection effects. Significant tests (p<0.05) are indicated in bold.
(PDF)

Table S5 Coefficients of variation (CVs) among genotypic means calculated separately for each environment (Fallow Field vs. Mowed Lawn), and in monoculture versus mixture, shown for both leaf area (cm^2^) and seed number variables.
(PDF)
