# Supplementary material for: Genotypic Diversity Effects on the Performance of Taraxacum officinale Populations Increase with Time and Environmental Favorability
Source: PLoS One. 2012 Feb 10;7(2):e30314. doi: 10.1371/journal.pone.0030314 (PMC3277588; doi:10.1371/journal.pone.0030314)

Figure S1. Comparison of genotypic performance in mixture versus monoculture over time. Dandelion genotype mixture (plot genotypic richness > 1) versus monoculture (plot genotypic richness =1) means ± 1 SE for leaf area (cm^2) at each of six measurement dates (N = 1800). Means are shown separately for (A) the fallow field and (B) the mowed lawn. Leaf area measurements were log-transformed before the genotypic means were calculated. The dashed line indicates a 1:1 relationship. Numbers refer to specific genotypes.

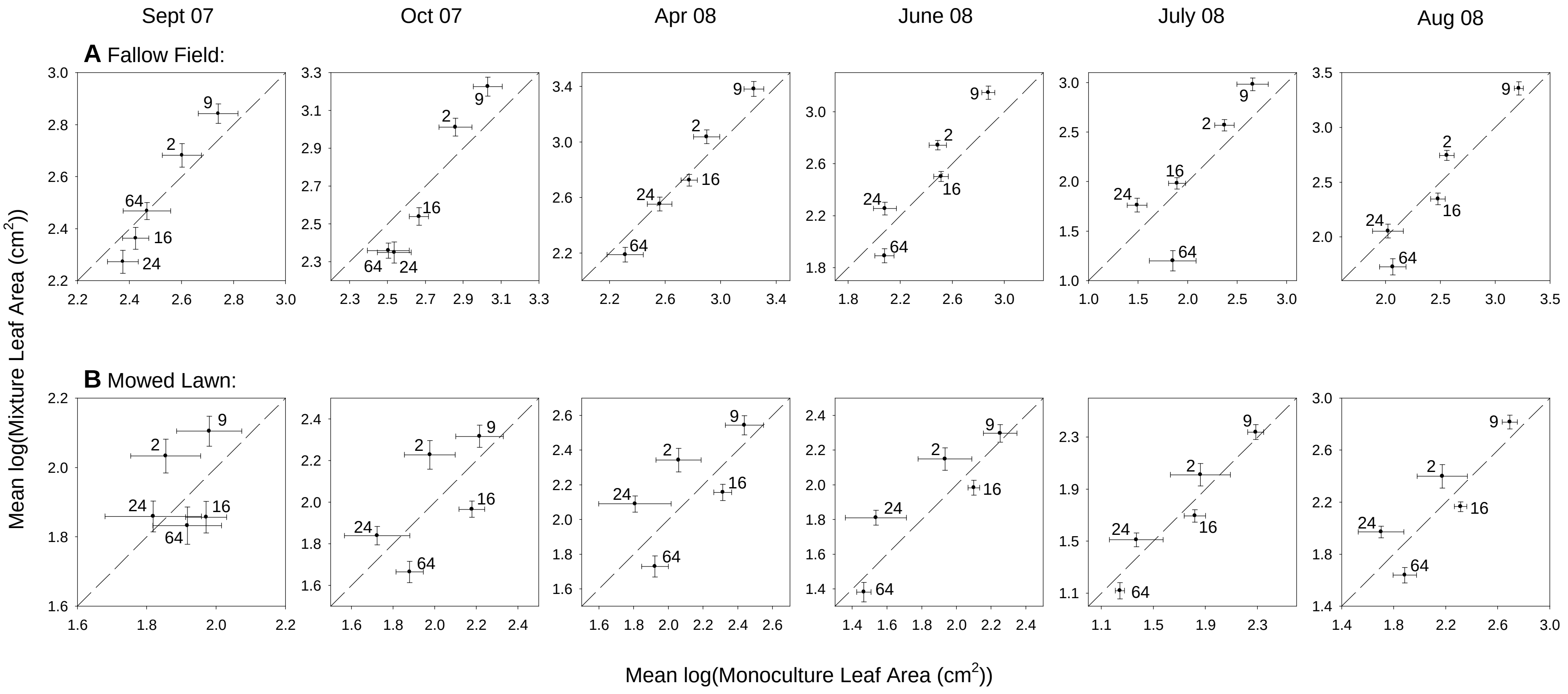

Supplement: Figure S1 — Comparison of genotypic performance in mixture versus monoculture over time. Dandelion genotype mixture (plot genotypic richness >1) versus monoculture (plot genotypic richness = 1) means ±1 SE for leaf area (cm2) at each of six measurement dates (N = 1800). Means are shown separately for a) the fallow field and b) the mowed lawn. Leaf area measurements were log-transformed before the genotypic means were calculated. The dashed line indicates a 1∶1 relationship. Numbers refer to specific genotypes. (PDF) [file pone.0030314.s001.pdf]
