# Supplementary material for: Genotypic Diversity Effects on the Performance of Taraxacum officinale Populations Increase with Time and Environmental Favorability
Source: PLoS One. 2012 Feb 10;7(2):e30314. doi: 10.1371/journal.pone.0030314 (PMC3277588; doi:10.1371/journal.pone.0030314)

Figure S2. Maximum number of emerged seedlings by genotype and environment. Mean of the maximum number of emerged seedlings (minus the number of emerged seedlings in a control) ± 1 SE for each genotype (genotypes are designated by numbers). Means are shown separately for (A) the fallow field and (B) the mowed lawn. There was a significant genotype-by-environment interaction (Mixed Model with Satterthwaite correction, $p = 0.02$), and significant main effects of genotype ($p = 0.01$) and environment ($p = 0.004$). Different letters indicate significant differences within an environment (Tukey-Kramer test, $p < 0.05$).

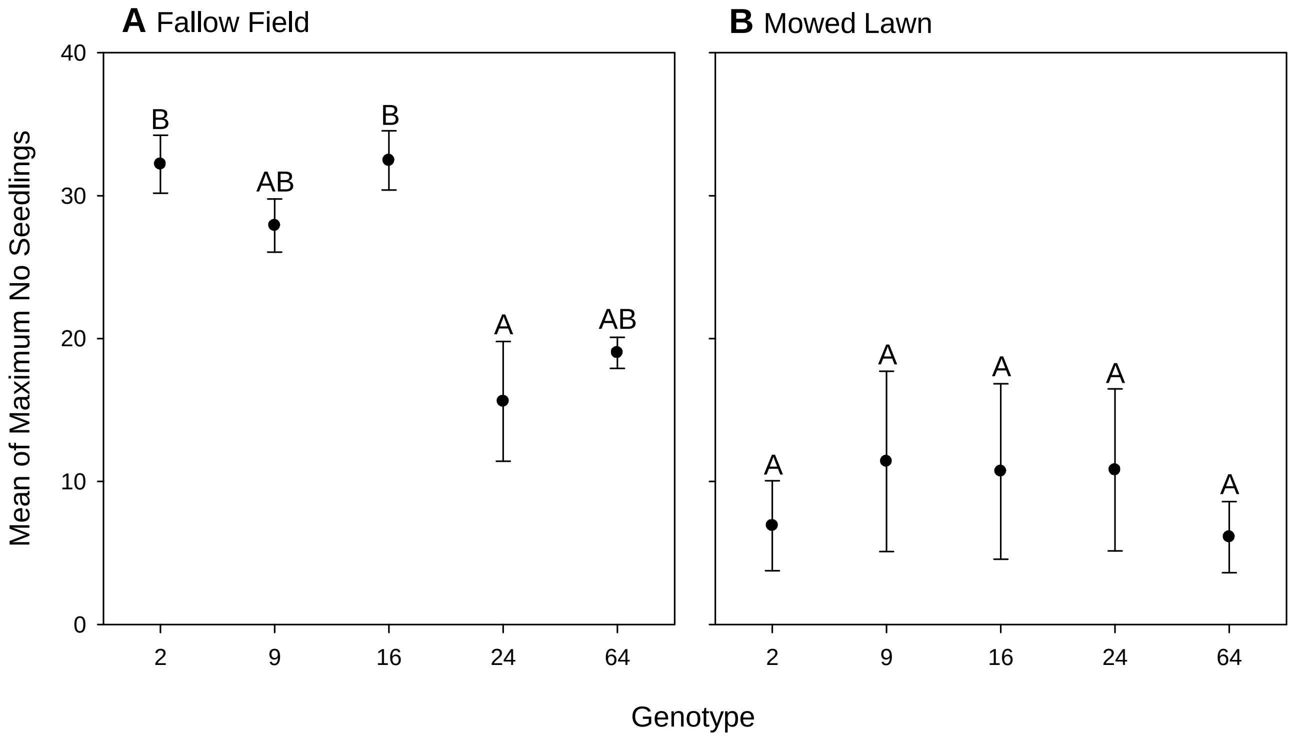

Supplement: Figure S2 — Maximum number of emerged seedlings by genotype and environment. Mean of the maximum number of emerged seedlings (minus the number of emerged seedlings in a control) ±1 SE for each genotype (genotypes are designated by numbers). Means are shown separately for (A) the fallow field and (B) the mowed lawn. There was a significant genotype-by-environment interaction (Mixed Model with Satterthwaite correction, p = 0.02), and significant main effects of genotype (p = 0.01) and environment (p = 0.004). Different letters indicate significant differences within an environment (Tukey-Kramer test, p<0.05). (PDF) [file pone.0030314.s002.pdf]
