# Supplementary material for: Genotypic Diversity Effects on the Performance of Taraxacum officinale Populations Increase with Time and Environmental Favorability
Source: PLoS One. 2012 Feb 10;7(2):e30314. doi: 10.1371/journal.pone.0030314 (PMC3277588; doi:10.1371/journal.pone.0030314)
Supplement: Table S1 — Description of diversity treatments as completed. The numbers in the composition column refer to specific genotypes. (PDF) [file pone.0030314.s003.pdf]

Table S1. Description of diversity treatments as completed. The numbers in the composition column refer to specific genotypes.

Genotypic Richness	Genotypic Composition	No Replicates
1	2	5
	9	5
	16	15
	24	5
	64	5
2[a]	2+16	10
	9+24	5
	9+64	5
	24+64	5
4	2+9+16+24	5
	2+9+16+64	5
	2+16+24+64	5
	9+16+24+64	5
5	2+9+16+24+64	10

[a]For treatments including two or four genotypes, genotypes were selected randomly with the constraint that each genotype be represented equally at each richness level.
