# Supplementary material for: Genotypic Diversity Effects on the Performance of Taraxacum officinale Populations Increase with Time and Environmental Favorability
Source: PLoS One. 2012 Feb 10;7(2):e30314. doi: 10.1371/journal.pone.0030314 (PMC3277588; doi:10.1371/journal.pone.0030314)
Supplement: Table S2 — The effects of environment, diversity (i.e. genotypic richness), and their interaction, as well as a covariate, prin1, the first axis of a principal components analysis that represented spatial variability in the edaphic environment, on multiple measures of population performance (summed across all plants in a plot): leaf area (cm2), seed number, seed-head number and aboveground biomass. Separate analyses of covariance (ANCOVAs) were performed for each variable and date. (PDF) [file pone.0030314.s004.pdf]

Table S2. The effects of environment, diversity (i.e. genotypic richness), and their interaction, as well as a covariate, prin1, the first axis of a principal components analysis that represented spatial variability in the edaphic environment, on multiple measures of population performance (summed across all plants in a plot): leaf area (cm^2), seed number, seed-head number and aboveground biomass. Separate analyses of covariance (ANCOVAs) were performed for each variable and date.

Variable	Date	Transformation	Effect	F	df	p
Plot Leaf Area (cm^2)	Sept 07	pwr(0.16)[a]	prin1	64.66	1, 173	< **0.0001**
			environment	321.39	1, 173	< **0.0001**
			diversity	2.98	2, 173	0.0534
			environment x diversity	0.96	2, 173	0.3856
	Oct 07	pwr(0.11)	prin1	59.52	1, 173	< **0.0001**
			environment	310.00	1, 173	< **0.0001**
			diversity	3.52	2, 173	**0.0318**
			environment x diversity	1.72	2, 173	0.1816
	Apr 08	pwr(0.10)	prin1	37.70	1, 173	< **0.0001**
			environment	175.11	1, 173	< **0.0001**
			diversity	4.50	2, 173	**0.0124**
			environment x diversity	0.97	2, 173	0.3816
	June 08	pwr(0.16)	prin1	24.45	1, 173	< **0.0001**
			environment	174.81	1, 173	< **0.0001**
			diversity	8.88	2, 173	**0.0002**
			environment x diversity	2.45	2, 173	0.0895
	July 08	pwr(0.18)	prin1	39.47	1, 173	< **0.0001**
			environment	42.12	1, 173	< **0.0001**
			diversity	13.07	2, 173	< **0.0001**
			environment x diversity	3.41	2, 173	**0.0352**
	Aug 08	pwr(0.13)	prin1	20.52	1, 173	< **0.0001**
			environment	42.02	1, 173	< **0.0001**
			diversity	9.70	2, 173	**0.0001**
			environment x diversity	1.36	2, 173	0.2587
Plot Seed No	Total 2007 + 2008	sqrt	prin1	37.85	1, 173	< **0.0001**
			environment	308.07	1, 173	< **0.0001**
			diversity	7.68	2, 173	**0.0006**
			environment x diversity	1.51	2, 173	0.2229

Table S2 (continued)

Plot Seed Head No	Total 2009 + 2010	pwr(0.31)	prin1	15.19	1, 173	**0.0008**
			environment	96.44	1, 173	**<0.0001**
			diversity	11.60	2, 173	**0.0004**
			environment x diversity	2.70	2, 173	0.1470
Plot Biomass	May 2010	pwr(0.38)	prin1	11.69	1, 173	**<0.0001**
			environment	43.98	1, 173	**<0.0001**
			diversity	3.58	2, 173	**0.0004**
			environment x diversity	0.63	2, 173	0.2376

[a]Pwr(x) indicates that the plot productivities were raised to the power of x.
