# Supplementary material for: Genotypic Diversity Effects on the Performance of Taraxacum officinale Populations Increase with Time and Environmental Favorability
Source: PLoS One. 2012 Feb 10;7(2):e30314. doi: 10.1371/journal.pone.0030314 (PMC3277588; doi:10.1371/journal.pone.0030314)
Supplement: Table S3 — Species recorded in the 2007 census of plot composition. Abundance classes were used for the principal components analysis. (PDF) [file pone.0030314.s005.pdf]

Table S3. Species recorded in the 2007 census of plot composition. Abundance classes were used for the principal components analysis.

Acer sp.
Achillea millefolium
Aphanes arvensis
Cerastium fontanum
Erodium cicutarium
Geranium molle
Geranium dissectum
Hypochaeris radicata
Lamium purpureum
Medicago lupulina
Montia linearis
Myosotis discolor
Plantago lanceolata
Poaceae[a]
Ranunculus repens
Rhytidiadelphus squarrosus
Rumex acetosella
Rumex obtusifolius
Sonchus asper
Spergularia rubra
Stellaria media
Taraxacum officinale
Trifolium spp.
Veronica arvensis

[a]Grass species were not discernable at this time of the year (June 2007).
