# Supplementary material for: Genotypic Diversity Effects on the Performance of Taraxacum officinale Populations Increase with Time and Environmental Favorability
Source: PLoS One. 2012 Feb 10;7(2):e30314. doi: 10.1371/journal.pone.0030314 (PMC3277588; doi:10.1371/journal.pone.0030314)
Supplement: Table S4 — Results of one-sample tests to determine whether mean net biodiversity, complementarity, or selection effects differed from zero. T-tests were used for net biodiversity and complementarity effects, and sign-tests for selection effects. Significant tests (p<0.05) are indicated in bold. (PDF) [file pone.0030314.s006.pdf]

Table S4. Results of one-sample tests to determine whether mean net biodiversity, complementarity, or selection effects differed from zero. T-tests were used for net biodiversity and complementarity effects, and sign-tests for selection effects. Significant tests ($p<0.05$) are indicated in bold.

			Fallow Field		Mowed Lawn	
Variable	Date	Effect	Transformation	p	Transformation	p
Leaf Area (cm²)	Sept 07	Net Effect	sqrt	**0.0497**	sqrt	0.8432
		Complementarity	pwr(0.75)[a]	0.1437	sqrt	0.6355
		Selection	none	**0.0027**	none	**0.0145**
	Oct 07	Net Effect	sqrt	**0.0007**	sqrt	0.7850
		Complementarity	pwr(0.75)	0.1201	sqrt	0.4378
		Selection	none	**< 0.0001**	none	**0.0065**
	Apr 08	Net Effect	sqrt	**0.0038**	sqrt	0.6467
		Complementarity	pwr(0.75)	0.0531	sqrt	0.0841
		Selection	none	**< 0.0001**	none	**0.0065**
	June 08	Net Effect	sqrt	**< 0.0001**	sqrt	0.5660
		Complementarity	pwr(0.75)	**< 0.0001**	sqrt	0.1458
		Selection	none	**0.0300**	none	**0.0300**
	July 08	Net Effect	sqrt	**0.0001**	sqrt	0.8268
		Complementarity	pwr(0.75)	**0.0003**	sqrt	0.6675
		Selection	none	0.1048	none	0.5900
	Aug 08	Net Effect	sqrt	**0.0005**	sqrt	0.1135
		Complementarity	pwr(0.75)	0.3152	sqrt	0.1365
		Selection	none	**0.0004**	none	0.5900
Seed Number	Total	Net Effect	none	**< 0.0001**	sqrt	0.2096
		Complementarity	none	0.0646	pwr(0.75)	**0.0063**
		Selection[b]	none	**< 0.0001**	none	0.2806

[a]Pwr(x) indicates that the data were raised to the power of x.
[b]A t-test was used in the fallow field.
