# Supplementary material for: Genotypic Diversity Effects on the Performance of Taraxacum officinale Populations Increase with Time and Environmental Favorability
Source: PLoS One. 2012 Feb 10;7(2):e30314. doi: 10.1371/journal.pone.0030314 (PMC3277588; doi:10.1371/journal.pone.0030314)
Supplement: Table S5 — Coefficients of variation (CVs) among genotypic means calculated separately for each environment (Fallow Field vs. Mowed Lawn), and in monoculture versus mixture, shown for both leaf area (cm2) and seed number variables. (PDF) [file pone.0030314.s007.pdf]

Table S5. Coefficients of variation (CVs) among genotypic means calculated separately for each environment (Fallow Field vs. Mowed Lawn), and in monoculture versus mixture, shown for both leaf area (cm^2) and seed number variables.

		Fallow Field		Mowed Lawn	
Variable	Date	Monoculture	Mixture	Monoculture	Mixture
Leaf Area (cm^2)	Sept 07	0.36	0.56	0.14	0.29
	Oct 07	0.52	0.93	0.45	0.64
	Apr 08	0.77	1.01	0.56	0.65
	June 08	0.77	1.09	0.68	0.66
	July 08	1.01	1.28	0.81	0.98
	Aug 08	1.22	1.44	0.82	1.02
Seed number	Total	0.48	1.00	0.59	0.78
